# Insights into RC time curve fit analysis of pulmonary artery pressure decay

**DOI:** 10.1186/s12890-024-03107-5

**Published:** 2024-06-25

**Authors:** Aristomenis Manouras, Lars H. Lund, Anikó Ilona Nagy, Jonas Johnson

**Affiliations:** 1 Heart and Vascular Center, Unit of Heart Failure, Arrhythmia and GUCH, Karolinska University Hospital, Stockholm, Sweden; 2 Department of Medicine, Solna Karolinska Institutet, 17177 Stockholm, Sweden; 3 Heart and Vascular Center, Semmelweis University, Budapest, Hungary; 4 Centre for Fetal Medicine Department of Obstetrics and Gynecology, Karolinska University Hospital, Stockholm, Sweden

**Keywords:** Pulmonary hypertension, Resistance, Capacitance, RC time

## Abstract

The notion of a constant relationship between resistance and capacitance (RC time) in the pulmonary circulation has been challenged by more recent research. The RC time can be obtained using either a simplified empirical approach or a semilogarithmic equation. Although direct curve-fit analysis is a feasible and ostensibly reference approach for RC analysis, it remains largely unexplored. We aimed to study the relationship between various RC methods in different states of pulmonary hemodynamics.

**Methods **In total, 182 patients underwent clinically indicated right heart catheterization. The pressure curves were exported and processed using the MATLAB software. We calculated the RC time using the empirical method (RC_EST_), semilogarithmic approach (RC_SL_), and direct measurement of curve fit (RC_FIT_).

**Results **Among 182 patients, 137 had pulmonary hypertension due to left heart disease (PH-LHD), 35 had pulmonary arterial hypertension (PAH), and 10 demonstrated normal hemodynamics (non-PH). RC_EST_ consistently overestimated the RC_FIT_ and RC_SL_ measurements by a mean of 75%. With all three methods, the RC values were longer in the PAH (RC_FIT_ = 0.36 ± 0.14 s) than in the PH-LHD (0.27 ± 0.1 s) and non-PH (0.27 ± 0.09 s) groups (*p* < 0.001). Although the RC_SL_ and RC_FIT_ values were similar among the three subgroups, they exhibited broad limits of agreement. Finally, the RC_EST_ demonstrated a strong discriminatory ability (AUC = 0.86, *p* < 0.001, CI = 0.79–0.93) in identifying PAH.

**Conclusion **RC time in PAH patients was substantially prolonged compared to that in PH-LHD and non-PH patients. The use of the empirical formula yielded systematic RC overestimation. In contrast, the semilogarithmic analysis provided reliable RC estimates, particularly for group comparisons.

**Supplementary Information:**

The online version contains supplementary material available at 10.1186/s12890-024-03107-5.

## Introduction

Within the context of pulmonary circulation, arterial load comprises both steady and pulsatile components. The steady element is governed by pulmonary vascular resistance (PVR), whereas the pulsatile component is regulated by pulmonary arterial compliance (PAC) through the elastic properties inherent to the pulmonary vasculature. Under diverse pulmonary hemodynamic conditions, resistance and compliance are inversely correlated in a hyperbolic manner. It has been postulated that the RC time, that is, the mathematical expression of the PAC and PVR relationship, which signifies the decay of pulmonary arterial pressure during diastole, remains constant across various states of pulmonary circulation [1–3]. The potential validity of RC constancy holds significant implications, rendering PVR and PAC measurements redundant, as knowledge of one of these measurements would facilitate the derivation of the other. However, subsequent research has revealed significant disparities in RC time across different states of pulmonary hemodynamics [4–6]. For instance, it has been shown that individuals with postcapillary pulmonary hypertension (PH) and those with normal pulmonary hemodynamics exhibit significantly shorter RC times than patients diagnosed with PAH [7].

The standard method for RC time derivation is based on an empirical approach that involves calculating the product of PVR and PAC, where PAC is determined by the ratio of stroke volume to pulmonary arterial pulse pressure (SV/PA_PP_). Importantly, this method does not reflect the exponential pressure decay during diastole and does not comply with the Windkessel function. Not surprisingly, it has been shown that this empirical RC time calculation overestimates the RC time [8]. To estimate the rate of diastolic pulmonary pressure drop more accurately, a semilogarithmic RC time derivation was first developed in animal studies by Engelberg and DuBois [9] and later employed in humans with cardiac and pulmonary diseases by Reuben [10]. To date, no direct comparison has been undertaken between the semilogarithmic and empirical methods for calculating RC time. However, the available observations suggest that the semilogarithmic approach yields significantly shorter RC values than the empirical method. Nevertheless, it is important to acknowledge that while the semilogarithmic method aligns better with the expression of the Windkessel function, the pressure fall during diastole may not always follow a complete monoexponential function, and a significant scattering of RC values along the curve may also occur.

Considering the aforementioned limitations of the available methods and the potential implications of RC analysis, an accurate evaluation of RC time could provide valuable information. It is feasible to directly assess the RC time by calculating the pulmonary pressure decay curve fit. Ideally, this approach allows for accurate determination of the RC time. However, to the best of our knowledge, direct RC measurements have not yet been undertaken.

The aim of the current study was to investigate the validity of both empirical and semilogarithmic approaches for RC calculation and to compare these two approaches with the hyperbolic curve fit of pulmonary artery pressure decay in different states of pulmonary circulation.

## Methods

### Study population

We prospectively evaluated patients who underwent right heart catheterization (RHC) at Karolinska University Hospital between February 2014 and August 2018. Patients were referred for RHC because of unexplained dyspnea or suspected pulmonary hypertension, or for hemodynamic assessment of heart failure for advanced treatment evaluation. Inclusion criteria were hemodynamically verified pulmonary hypertension (PH) i.e., mean pulmonary artery pressure > 20 mmHg. Patients with constrictive pericarditis, arrhythmogenic right ventricular cardiomyopathy, previous heart transplantation were excluded. Additionally, patients without a distinct dicrotic notch in the pulmonary artery tracings were excluded from the analysis. 10 patients with normal hemodynamics were included as control group.

All patients underwent transthoracic echocardiography within 2 h before RHC, following the current recommendations [11]. This study was approved by the regional ethical review board and complied with the Declaration of Helsinki. Informed consent was obtained from all participants.

### Right heart catheterization

All patients were in a stable hemodynamic condition during RHC, which was performed through jugular vein access using a 6F balloon-tipped fluid-filled Swan–Ganz catheter (Edwards Lifesciences, Irvine, California, USA). Pressure measurements were obtained under fluoroscopy after calibration with the zero-level set at the mid-thoracic line at the end-expiration during spontaneous breathing and stored in dedicated software (Xper Information Management, Philips Medical Systems, The Netherlands). Fick´s principle was used to assess cardiac output (CO). Oxygen consumption was measured breath-by-breath using a Jaeger Oxycon Pro (VIASYS Healthcare, Palm Springs, California, USA). The arteriovenous oxygen difference was calculated from the oxygen concentration in the arterial and mixed venous blood from the pulmonary artery. Thermodilution was performed in ten patients. As part of the hemodynamic evaluation, patients with clinically suspected HF, normal EF, and normal pulmonary wedge pressure at rest (PAWP_REST_ ≤ 15 mmHg) underwent supine cycle ergometry, as well as those with HF and reduced EF (HFrEF). Patients cycled at 60 rpm in the supine position for 1 min at 10 W before the workload was incrementally increased at 2-min intervals until maximal volitional exertion was achieved.

### Pressure calculations

The traces were stored and exported to an external hard drive and subsequently imported as text files into MATLAB (MATLAB software; R2018b, MathWorks, MA, USA). The data were then converted back to the original pressure tracings, and the ECG was simultaneously displayed using the built-in import function of MATLAB. Each heart cycle appropriate for analysis was manually selected based on two criteria: recordings at the end of expiration and adequate tracing quality. A graphical user interface (GUI) was designed and programmed in MATLAB, which enabled manual selection of specific points and regions of the curve for dedicated offline analysis of each selected cycle. This system allows the simultaneous display of both waveforms along with the corresponding ECG traces. First, the ECGs of the two recordings were synchronized manually to achieve optimal temporal harmonization, despite non-beat-to-beat measurements. From the PAP recordings, the peak of the ascending limb of the PAP curve (PAP_S_) and the end diastolic pressure (PAP_D_) were identified and marked manually, following which the software provided an automated calculation of PAP_S_ and PAP_D_. Subsequently, the mean PAP (PAP_M_) was calculated using PAP integration over the entire cardiac cycle.

**Fig. 1:**
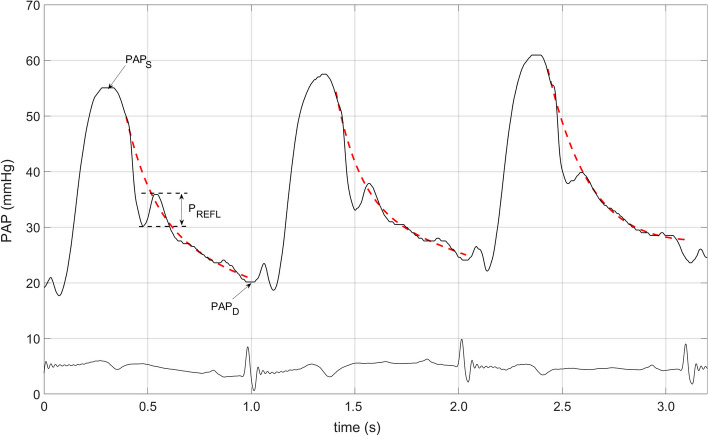
Pulmonary artery pressure recording and analysis. PAP_S_, peak systolic pulmonary artery pressure; PAP_D_, diastolic pulmonary artery pressure. P_REFL_, peak amplitude of the reflection wave; the red dotted line denotes the PAP curve fit

**Fig. 2:**
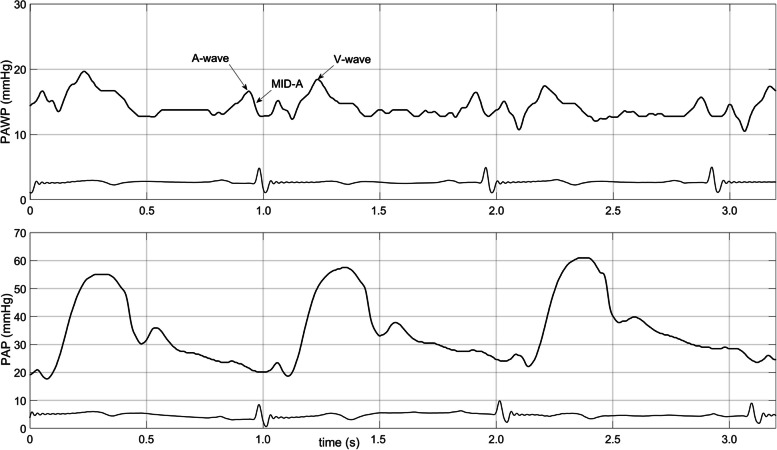
Pulmonary artery wedge (PAWP) and pulmonary artery pressure (PAP) recording. The tracings were synchronized manually using MATLAB software. The A-wave denotes the peak A-wave pressure, and the V-wave denotes the peak V-wave pressure. PAWP pressures were measured as mid-A-wave pressures

The peak amplitudes of the reflected waves were subsequently denoted and measured, as shown in Fig. 1. PAWP measurements in patients with sinus rhythm (SR) were performed at the mid-A-wave (PAWP_Mid-A_) [12] (Fig. 2), whereas in patients with atrial fibrillation (AF), the pressure was recorded 130 ms after QRS onset [12]. Subsequently, the PVR and DPG were calculated using the following equations:

$$\text{PVR }=\left({\text{PAP}}_{\text{M}}-\text{ PAWP}\right)/\text{CO}$$

$$\text{DPG }= {\text{PAP}}_{\text{D}}-\text{PAWP}$$

An abnormal PAWP response during exercise was defined as a PAWP_EX_≥ 25 mmHg [13].

Pulmonary hypertension due to left heart disease (PH-LHD) was defined as elevated mean pulmonary artery pressure (PAP_M_ > 20 mmHg) and PAWP_REST_ > 15 mmHg or PAWP_EX_ ≥ 25 mmHg during exertion. Within PH-LHD, two groups were subsequently identified: those with PVR < 2 WU, denoted as isolated postcapillary PH (IpC-PH), and those with PVR ≥ 2 WU, defined as combined pre- and postcapillary PH (CpC-PH).

Pulmonary arterial hypertension (PAH) was defined as PAP_M_ > 20 mmHg, PAWP_REST_ < 15 mmHg, PVR > 2 WU, PAWP_EX_< 25 mmHg, and consensus clinical board opinion [12–14].

### RC measurements

The RC time was calculated using the following three approaches.

1. RC time estimation using the empirical approach: RC_EST_ = PAC × PVR × 0.06, sec. PAC was calculated as the ratio of stroke volume to pulmonary arterial pulse pressure (SV/PA_PP_).
2. RC time measurements based on the semilogarithmic formula [15]. Diastolic pulmonary arterial pressure can be expressed as a single exponential function:

1 $$P\left(t\right)={(P}_{0}-{P}_{\infty })*{e}^{-\frac{t}{RC}}+{P}_{\infty }$$

where ${P}_{0}$ refers to the notch pressure in the PAP curve and RC is the time constant, while ${P}_{\infty }$ denotes the zero-flow pressure at infinite time, which is approximated by the wedge pressure. To calculate the RC time, the log transformation of Eq. 1 is employed:

2 $$RC=\frac{DT}{\mathit{ln}{ (P}_{0}-{P}_{PAWP})/({PAP}_{D}-{P}_{PAWP})}$$

where DT denotes the diastolic time, i.e., the time between the pressure at the notch and the diastolic pulmonary pressure, PAP_D_ denotes the end-diastolic pulmonary pressure, and P_PAWP_ denotes the wedge pressure.

3. RC time derived from curve fit (RC_FIT_). MATLAB software was used to estimate the nonlinear fit. The starting point of the fit was set immediately following the dichrotic notch passing through the end-diastolic PAP, and the asymptote was set at the calculated PAWP, as described above (Fig. 1). The fit was based on the Levenberg–‒Marquardt nonlinear least-squares algorithm and followed the exponential function provided by Eq. 1. The RC time was calculated at the point of the curve at which the starting pressure decreased by 63%.

PAC derived from RC_SL_ and RC_FIT_ were adjusted to estimate PAC in mL/mmHg by multiplying the ratios by 16.67 (PAC_SL_ = (RC_SL_/PVR) * 16.67, PAC_FIT_ = (RC_FIT_/PVR) * 16.67).

To ensure uniformity in data acquisition and analysis, the same investigator (AM) participated in most RHC procedures and analyzed all waveforms while blinded to patient data. All measured pressures were analyzed using 1–4 heart cycles, depending on the quality of the pressure recording, and then averaged.

### Statistical analysis

Normality was assessed using the Shapiro–Wilk test, and statistical analyses were performed using SPSS (version 23.0; SPSS Inc., Chicago, IL, USA). Continuous variables were compared using the Mann–Whitney U test for skewed variables. All tests were performed with a 95% confidence interval, and measurements are presented as the median and interquartile range (IQ) or mean and standard deviation (SD), as appropriate. Correlations were evaluated using Spearman's rho or Pearson's two-tailed tests. Intraobserver and interobserver reproducibility of instantaneous PAWP measurements was assessed using the intraclass correlation coefficient (ICC) in ten randomly selected patients. Survival was analyzed using the Kaplan–Meier nonparametric test and compared using the log-rank test. For survival analysis, patients who underwent cardiac transplantation or ventricular assist device implantation were censored at the time of the event. The quality of the curve fit was assessed using the mean square error (MSE) of the fitted model and was returned as a scalar value. MSE is an estimate of the variance in the error term. The diagnostic ability of various PAWP measurements was assessed using receiver operating characteristic (ROC) analysis, and ROC curves were compared using the DeLong test. Statistical significance was set at *p* < 0.05. Cox-regression analysis was employed for prognostic evaluation.

## Results

### Patient characteristics

Baseline clinical and hemodynamic characteristics of the study cohort are shown in Table 1. A total of 249 patients were screened. 194 patients fulfilled the eligibility criteria and were included in this study. In 12 cases, the dicrotic notch could not be identified with certainty and thus was not included in the analysis. In effect, 182 patients were analyzed. Of these, 137 were classified as PH-LHD, 35 as PAH, and the ten controls as non-PH. Among the PH-LHD patients, 77 (56%) were classified as HFpEF [EF = 62%; IQR:56–65] and 60 (44%) as HFrEF [EF = 26%; IQR:19–42]. 17 (28%) of the HFrEF patients were on resynchronization therapy. 78% of the PH-LHD and PAH groups presented New York Heart Association (NYHA) classes III-IV. AF was recorded in 66 patients during the examination. In total, 485 curves from the 182 study patients were analyzed (four heart cycles in 17 patients, three in 94 patients, two in 63 patients, and one in 8 patients).

**Table 1:** Demographic and echocardiographic characteristics of the three subgroups of the study cohort

Demographics	PH-LHD (*n* = 137)	PAH (*n* = 35)	Non-PH (*n* = 10)
**Age** (years)	63 ± 15 [137]	57 ± 15 [35]	61 ± 16 [10]
**Female** (n,%)	60 (44)	20 (57)	7 (70)
**LVEF** < 50% (%)	56	—	—
**AF** (%)	66 (48)	0 (0)	0 (0)
**Diabetes mellitus** (%)	15	6	10
**Hypertension** (%)	56	15	60
**BMI** (kg/m^2^)	27.1 ± 5.7 [137]	25.8 ± 5.6 [35]	24.4 ± 3.9 [10]
**EF** (%)	47.4 ± 19 [137]	61.1 ± 6.4 [34]	62.8 ± 5.5 [10]
**Hemodynamic data**			
**SBP** (mmHg)	117.8 ± 27.2 [132]	116.9 ± 21.4 [34]	130.8 ± 13 [10]
**DBP** (mmHg)	65.8 ± 14.1 [132]	68.2 ± 11.4 [34]	68.7 ± 12.4 [10]
**CI** (L/min/m^2^)	2.5 ± 0.9 [137]**	2.5 ± 0.67 [35]†	3.4 ± 0.7 [10]
**HR** (beats/min)	70.1 ± 11.8 [137]	73.1 ± 12.6 [35]	72 ± 8 [10]
**PAP**_**M**_ (mmHg)	33 ± 9.8 [137]**‡‡	42 ± 13.5 [35]††	17.7 ± 2 [10]
**PAP**_**D**_ (mmHg)	21.5 ± 7.1 [137]**‡	27.2 ± 11.5 [35]††	10 ± 2 [10]
**PAWP** (mmHg)	18.2 ± 5.1 [113]**‡‡	9.6 ± 3.5 [34]	8.5 ± 1.8 [10]
**PVR** (WU)	3.5 ± 2.3 [137]*‡‡	7.8 ± 4.3 [35]††	1.6 ± 0.6 [10]
**PAC** (mL/mmHg)	2.7 ± 1.4 [137]**‡‡	1.81 ± 0.9 [35]††	4.5 ± 1 [10]
**DPG** (mmHg)	3.1 ± 5.8 [137]‡‡	17.6 ± 11.8 [35]††	1.5 ± 1.9 [10]
**RC**_**EST**_ (sec)	0.42 (0.33–0.52) [137]‡‡	0.68 (0.53–0.78) [35]††	0.39 (0.36–0.47) [10]
**RC**_**SL**_ (sec)	0.28 (0.21–0.40) [112]‡‡	0.45 (0.35–0.57) [35]††	0.23 (0.17–0.29) [9]
**RC**_**FIT**_ (sec)	0.26 (0.19–0.33) [132]‡‡	0.33 (0.28–0.48) [22]	0.27(0.20–0.32) [10]

*PH-LHD* Pulmonary hypertension in left heart disease, *PAH* Pulmonary arterial hypertension, *Non-PH* No pulmonary hypertension, *EF* Ejection fraction, *AF* Atrial fibrillation, *BMI* Body mass index, *SBP* Systolic blood pressure, *DBP* Diastolic blood pressure, *CI* Cardiac index, *HR* Heart rate, *PAP*_*M*_ Mean pulmonary artery pressure, *PAP*_*D*_ Diastolic pulmonary artery pressure, *PAWP* Pulmonary artery wedge pressure, *PVR* Pulmonary vascular resistance, *PAC* Pulmonary arterial capacitance, *DPG* Diastolic pressure gradient, *RC*_*EST*_ RC derived from the empirical approach, *RC*_*SL*_ RC derived from the semilogarithmic equation, *RC*_*FIT*_ RC derived from curve fit analysis

^*^signifies a significant difference between PH-LHD and non-PH at a level of *p* < 0.05

^**^p < 0.001 between PH-LHD and non-PH

^‡‡^*p* < 0.001 between PH-LHD and PAH

^†^signifies *p* < 0.05 between PAH and non-PH

^††^signifies *p* < 0.001 between PAH and non-PH

### RC measurements

Considering the entire cohort, RC_EST_ exhibited longer values (0.44; 0.36–0.58 s) compared to the RC_FIT_ (0.27; 0.2–0.35 s) and RC_SL_ (0.32; 0.23–0.42 s), *p* < 0.001 for both. In contrast, the difference between RC_FIT_ and RC_SL_ was not significant (*p* = 0.95).

As shown in Table 1, the RC values measured using each of the three approaches were longer in the PAH group than in the PH-LHD and non-PH groups (*p* < 0.001). In contrast, the RC did not differ significantly between the PH-LHD and non-PH groups (*p* > 0.05) for each of the three RC analysis. Within the PH-LHD group, IpC-PH patients (*n* = 37) had significantly shorter RC times than the CpC-PH subgroup (*n* = 100) for each of the three methods (RC_EST_ = 0.32;0.18–0.4 s vs.0.45; 0.38–0.56 s; RC_SL_ = 0.23; 0.20–0.25 s vs. 0.33; 0.22–0.41 s; RC_FIT_ = 0.21; 0.14–0.29 s vs. 0.28; 0.2–0-36 s; *p* < 0.001 in all).

### Hemodynamic determinants of the RC estimates

As shown in Table 2, for the entire cohort, DPG exhibited the most robust association with each of the three RC approaches as compared to PVR, PAWP, CO and HR. Multivariate analysis demonstrated that DPG (*p* < 0.001) and HR (*p* < 0.001) were the only significant predictors of RC time derived using each of the three approaches whereas for the RC_EST_, PAWP was identified as an additional significant determinant (*p* = 0.003).

**Table 2:** Correlations between RC measurements and hemodynamic variables

		**RC**_**FIT (n)**_	**RC**_**SL**_** (n)**	**RC**_**EST**_** (n)**
**Entire Cohort**	**RC**_**FIT**_		0.65** (138)	0.69** (164)
	**RC**_**SL**_	0.65** (138)		0.86** (156)
	**RC**_**EST**_	0.69** (164)	0.86** (156)	
	**PVR**	0.36** (164)	0.61** (156)	0.61** (182)
	**DPG**	0.48** (164)	0.76** (156)	0.76** (182)
	**PAWP**	-0.35** (141)	-0.34** (135)	-0.49** (157)
	**HR**	-0.25** (164)	-0.16* (156)	-0.14 (182)
	**PAP**_**D**_	0.14 (164)	0.50** (156)	0.46** (182)
	**CO**	-0.16* (164)	-0.18* (156)	-0.08 (182)
**LHD-PH**			0.58** (107)	0.61** (132)
		0.58** (107)		0.75** (112)
		0.61** (132)	0.75** (112)	
	**PVR**	0.31** (132)	0.37** (112)	0.48** (137)
	**DPG**	0.46** (132)	0.68** (112)	0.73** (137)
	**PAWP**	-0.28** (109)	-0.11 (92)	-0.32** (113)
	**HR**	-0.13 (132)	-0.25** (112)	-0.20* (137)
		0.13 (132)	0.33** (112)	0.32** (137)
	**CO**	-0.25** (132)	-0.15 (112)	-0.06 (137)
**PAH**			0.83** (22)	0.89** (22)
		0.83** (22)		0.92** (35)
		0.89** (22)	0.92** (35)	
	**PVR**	0.19 (22)	0.60** (35)	0.44** (35)
	**DPG**	0.22 (22)	0.67** (35)	0.59** (35)
	**PAWP**	-0.33 (22)	-0.35* (34)	-0.35* (34)
	**HR**	-0.78** (22)	-0.19 (35)	-0.22 (35)
		0.06 (22)	0.59** (35)	0.50** (35)
	**CO**	0.06 (22)	-0.31 (35)	-0.1 (35)
**Non-PH**			0.33 (9)	0.53 (10)
		0.33 (9)		0.76* (9)
		0.53 (10)	0.76* (9)	
	**PVR**	0.18 (10)	0.35 (9)	0.74* (10)
	**DPG**	0.35 (10)	0.88** (9)	0.84** (10)
	**PAWP**	-0.92** (10)	-0.48 (9)	-0.55 (10)
	**HR**	-0.51 (10)	-0.45 (9)	-0.78** (10)
		-0.47 (10)	0.3 (9)	0.32 (10)
	**CO**	0.53 (10)	-0.15 (9)	-0.21 (10)

RC_EST_ denotes RC-derived using the empirical approach, *RC*_*SL*_ RC derived from the semilogarithmic equation, *RC*_*FIT*_ RC derived from the curve fit analysis, *PVR* Pulmonary vascular resistance, *DPG* Diastolic pressure gradient, *PAWP* Pulmonary artery wedge pressure, *HR* Heart rate, *PAP*_*D*_ Diastolic pulmonary artery pressure, *CO* Cardiac output, *PH-LHD* Pulmonary hypertension in left heart disease, *PAH* Pulmonary arterial hypertension, *Non-PH* No pulmonary hypertension

^*^signifies a significant difference at a level of *p* < 0.05

^**^signifies a significant difference at a level of *p* < 0.001

To identify the predictors of RC in each of the three patient groups, separate multivariate analyses was conducted using DPG, PAWP, HR, and CO as independent variables, as illustrated in Table 3. As provided, in the PH-LHD group, regression analysis demonstrated that DPG, PAWP, HR, and CO were significantly associated with RC_FIT_ with the model explaining 32% of the logRC_FIT_ variance (*R*^2^ = 0.32, F (4,127) = 15, *p* < 0.001). In contrast, in PAH group, only DPG and HR, acted hierarchically as independent predictors (*R*^2^ = 0.68, F (4,17) = 9.0, *p* < 0.001). In the non-PH subgroup, PAWP was significantly associated with RC_FIT_.

**Table 3:** Multivariable regression analysis of the determinants of various RC analyses

	**RC**_**EST**_				**RC**_**SL**_				**RC**_**FIT**_			
	B	SE	Beta	P	B	SE	Beta	P	B	SE	Beta	P
**PH-LHD**												
**DPG**	0.02	0.002	0.72	** < 0.001**	0.02	0.002	0.65	** < 0.001**	0.014	0.003	0.384	** < 0.001**
**PAWP**	-0.007	0.001	-0.20	** < 0.001**	-0.004	0.002	-0.14	**0.04**	-0.008	0.003	-0.23	**0.003**
**HR**	-0.003	0.001	-0.20	** < 0.001**	-0.003	0.001	-0.28	** < 0.001**	-0.002	0.001	-0.16	**0.04**
**CO**	0.005	0.005	0.05	0.38	-0.012	0.007	-0.13	0.06	-0.02	0.008	-0.22	**0.004**
**PAH**												
**DPG**	0.007	0.001	0.62	** < 0.001**	0.009	0.001	0.65	** < 0.001**	0.008	0.004	0.32	**0.05**
**PAWP**	-0.01	0.004	-0.27	**0.04**	-0.009	0.006	-0.19	0.12	-0.001	0.008	-0.03	0.84
**HR**	-0.003	0.001	-0.35	**0.01**	-0.004	0.002	-0.32	**0.01**	-0.01	0.002	-0.79	** < 0.001**
**CO**	0.008	0.01	0.09	0.49	-0.015	0.01	-0.12	0.30	0.012	0.02	0.1	0.54
**Non-PH**												
**DPG**	0.028	0.01	0.5	**0.03**	0.09	0.023	0.84	**0.02**	-0.005	0.013	-0.06	0.7
**PAWP**	-0.021	0.01	-0.34	0.13	-0.006	0.03	-0.06	0.85	-0.063	0.017	-0.66	**0.01**
**HR**	-0.005	0.003	-0.34	0.12	-0.008	0.008	-0.31	0.36	-0.009	0.0036	-0.42	0.06
**CO**	-0.015	0.014	-0.19	0.33	0.048	0.04	0.39	0.31	0.03	0.02	0.25	0.18
**All**												
**DPG**	0.014	0.001	0.67	** < 0.001**	0.014	0.001	0.68	** < 0.001**	0.01	0.002	0.37	** < 0.001**
**PAWP**	-0.006	0.002	-0.22	** < 0.001**	-0.003	0.002	-0.11	**0.045**	-0.001	0.002	-0.22	** < 0.001**
**HR**	-0.003	0.0006	-0.22	** < 0.001**	-0.004	0.0007	-0.27	** < 0.001**	-0.003	0.001	-0.24	** < 0.001**
**CO**	0.003	0.005	0.03	0.52	-0.013	0.006	-0.11	**0.042**	-0.017	0.008	-0.15	**0.03**

RC_EST_ denotes RC derived using the empirical approach, *RC*_*SL*_ RC derived from the semilogarithmic equation, and RC_FIT_, RC derived from the curve fit analysis. *PH-LHD* Pulmonary hypertension in left heart disease, *PAH* Pulmonary arterial hypertension, *Non-PH* no pulmonary hypertension, *DPG* Diastolic pressure gradient, *PAWP* Pulmonary artery wedge pressure, *HR* Heart rate, *CO* Cardiac output, *SE* Standard error, *B* Unstandardized coefficient Beta, standardized coefficient, *Sig* significance

In regard to RC_SL_, regression analysis demonstrated DPG, PAWP, and HR as predictors in the PH-LHD group, together explaining 55% of the logRC_SL_ variance (*R*^2^ = 0.55, F (4,107) = 32, *p* < 0.001). In the PAH group, only DPG and HR were independent predictors (*R*^2^ = 0.61, F (4,30) = 11.6, *p* < 0.001). Finally, in the non-PH subgroup, only the DPG was significantly associated with RC_SL_.

Finally, for RC_EST_, in the PH-LHD group and PAH group, DPG, PAWP, and HR were significant predictors (*R*^2^ = 0.65, F (4,132) = 62, *p* < 0.001) and (*R*^2^ = 0.56, F (4,30) = 9.6, *p* < 0.001) respectively. Notably, in non-PH, only DPG was associated with RC_EST_.

### RC-time by the empirical vs. the curve fit approach

**Fig. 3:**
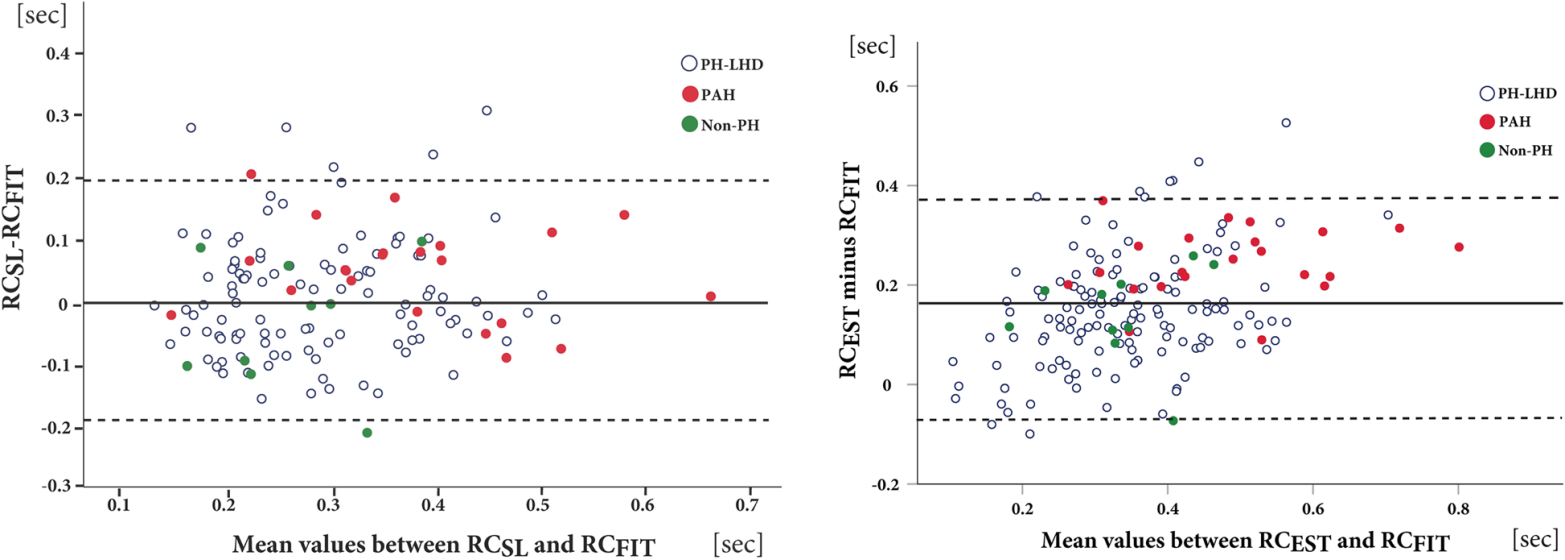
Bland–Altman plots between the RC values derived from the semilogarithmic approach and the curve fit analysis (left panel), and between the RC calculated using the empirical formula and curve fit analysis (right panel). RC_EST_ denotes the RC values calculated using the empirical formula, RC_FIT_; the RC values measured by curve fit analysis and RC_SL_ the RC values calculated using the semilogarithmic approach Non-PH, subjects without pulmonary hypertension; PAH, pulmonary arterial hypertension; PH-LHD, pulmonary hypertension due to left heart disease

Despite a significant correlation with both RC_SL_ and RC_FIT_, the RC_EST_ yielded significantly higher values than the other two methods in all three patient groups (*p* < 0.001; Tables 1 and 2). As demonstrated in Fig. 3, RC_EST_ systematically overestimated RC_FIT_ values by an average of 76% (mean 0.155 s; limits of agreement (LOA): − 0.07–0.38 s. The extent of overestimation (*Δ*RC_EST-FIT_) was comparable for the three subgroups: PAH (*Δ*RC_EST-FIT_ = mean 0.24 s [84%], LOA = 0.1–0.38), PH-LHD (*Δ*RC_EST-FIT_ = 0.14 s [75%], LOA =  − 0.08–0.36), and non-PH (*Δ*RC_EST-FIT_ = 0.16 s [75%], LOA =  − 0.02–0.34).

For the entire cohort, DPG emerged as the strongest predictor of the difference between RC_EST_ and RC_FIT_ (*Δ*RC_EST-FIT_) (β = 0.56, *p* < 0.001), whereas weaker yet significant associations were observed for CO (β = 0.19, *p* = 0.01) and HR (β =  − 0.15, *p* = 0.034). As shown in Table 4, in the PH-LHD subgroup, DPG more accurately predicted *Δ*RC_EST-FIT_, with CO and HR exhibiting weaker associations. In the PAH subgroup, only PAWP displayed a significant inverse association with *Δ*RC_EST-FIT_, whereas in the non-PH subgroup, DPG was strongly associated with *Δ*RC_EST-FIT_.

**Table 4:** Multivariable regression analysis for the difference between RC_SL_ and RC_FIT_ (upper panel), and the respective analysis for the difference between RC_EST_ and RC_FIT_ (lower panel)

	B	SE	β	p
**Multivariable Linear regression for ***Δ***RC**_**SL-FIT**_				
**PH-LHD**				
DPG	0.007	0.002	0.35	**0.001**
PAWP	0.001	0.002	0.03	0.78
HR	-0.001	0.001	-0.11	0.29
CO	0.002	0.006	0.03	0.78
**PAH**				
DPG	0.003	0.002	0.24	0.25
PAWP	-0.005	0.005	-0.25	0.25
HR	0.003	0.001	0.47	**0.028**
CO	-0.012	0.01	-0.23	0.25
**Non-PH**				
DPG	0.05	0.02	0.85	**0.023**
PAWP	0.03	0.02	0.42	0.24
HR	0.001	0.005	0.12	0.71
CO	-0.012	0.03	-0.17	0.66
**Multivariable Linear regression for *Δ*RC**_**EST-FIT**_				
**PH-LHD**				
DPG	0.007	0.002	0.35	**0.001**
PAWP	0.001	0.002	0.03	0.77
HR	-0.001	0.001	-0.11	0.29
CO	0.002	0.006	0.03	0.77
**PAH**				
DPG	0.003	0.002	0.24	0.25
PAWP	-0.005	0.005	-0.25	0.25
HR	0.003	0.001	0.47	**0.03**
CO	-0.013	0.01	-0.23	0.25
**Non-PH**				
DPG	0.05	0.02	0.83	**0.023**
PAWP	0.025	0.018	0.42	0.24
HR	0.002	0.001	0.12	0.71
CO	-0.01	0.026	-0.17	0.66

RC_EST_ denotes RC-derived using the empirical approach, *RC*_*SL*_ RC derived from the semilogarithmic equation, *RC*_*FIT*_ RC derived from the curve fit analysis, *DPG* Diastolic pressure gradient, *PAWP* Pulmonary artery wedge pressure, *HR* Heart rate, *PAP*_*D*_ Diastolic pulmonary artery pressure, *CO* Cardiac output. B, unstandardized coefficient, *SE* Standard error, *β* Standardized coefficient, *p* level of significance

### RC-time by the empirical vs. the semilogarithmic approach

RC_EST_ systematically overestimated RC_SL_ values by an average of 63% (mean 0.18 s; limits of agreement (LOA): − 0.008–0.37 s. The extent of overestimation (*Δ*RC_EST-FIT_) for the three subgroups was: PAH (*Δ*RC_EST-SL_ = mean 0.21 s [ 50%], LOA = 0.03–0.39), PH-LHD (*Δ*RC_EST-SL_ = 0.17 s [65%], LOA =  − 0.022–0.36), and non-PH (*Δ*RC_EST-SL_ = 0.18 s [91%], LOA =  − 0.06–0.30).

A multivariable linear analysis was conducted to identify the determinants of the difference between RC_EST_ and RC_SL_ (*Δ*RC_EST-SL_). For the entire cohort, the DPG (β = 0.21, *p* < 0.022) and CO (β = 0.22, *p* = 0.008) were significant predictors of *Δ*RC_EST-SL_. In contrast, HR (*p* = 0.07) and PAWP (*p* = 0.14) were not significantly associated with the *Δ*RC_EST-SL_.

### RC time using the semilogarithmic vs. curve fit approach

For the entire cohort, the RC_SL_ was moderately associated with the corresponding RC_FIT_ values (*r* = 0.62, *p* < 0.001) with the relationship being stronger in the PAH group (*r* = 0.83, *p* < 0.001) compared to the PH-LHD group (*r* = 0.58, *p* < 0.001); No association was observed in the non-PH group (*p* > 0.13) (Table 2). Although the RC_SL_ and RC_FIT_ values did not differ significantly (RC_SL_ = 0.33; 0.23–0.42 s; RC_FIT_ = 0.27, 0.2–0.35 s, *p* = 0.54), the limits of agreement were broad in pairwise comparisons (*Δ*RC_SL-FIT_ 0.009 ± 0.095 s) (Fig. 3).

Similar were the findings in the subgroup analysis: PH-LHD (*Δ*RC_SL-FIT_ = 0.005; *Δ*% = 14%; LOA =  − 0.199 to 0.201) and non-PH (*Δ*RC_SL-FIT_ =  − 0.04; *Δ*% = 8%; LOA =  − 0.196 to 0.204) and even larger in the PAH subgroup (*Δ*RC_SL-FIT_ = 0.05 s; *Δ*% = 20%, LOA =  − 0.11 − 0.21).

Of the 485 analyzed curves, paired comparisons between RC_SL_ and RC_FIT_ were obtained in 297 cases. The mean RC_SL_ (0.296 ± 0.12 s) and RC_FIT_ (0.30 ± 0.12 s) values were near identical, with ΔRC_SL__-__FIT_ =  − 0.003 ± 0.1 s. RC_SL_ > RC_FIT_ was observed in 49% of the cases. Patients with RC_SL_ > R_FIT_ had higher DPG (7.9 ± 6.9 vs. 3.4 ± 4 mmHg), PVR (4.8 ± 3 vs. 3.4 ± 1.9 WU), and PA_PP_ (36 ± 16 vs. 27 ± 11 mmHg; *p* < 0.001 for all).

Multivariable linear regression analysis in the entire cohort, demonstrated DPG as the only predictor of the difference between RC_SL_ and RC_FIT_ (ΔRC_SL__-__FIT_) (β = 0.41, *p* < 0.001). In the LHD and non-PH groups, DPG was the only significant predictor of ΔRC_SL__-__FIT_. However, for the PAH group, the HR only acted as a ΔRC_SL-FIT_ determinant (Table 4).

### Feasibility and variability of the RC measurements

RC_EST _calculation was feasible for all 182 patients. As inferred from Eq [16], the RC_SL_ calculation requires PAP_D_ being greater than the PAWP. In total, 373 of the 485 curves (77%) were suitable for the RC_SL_ calculations, corresponding to 156 patients (86%). Similarly, the RC_FIT_ calculation requires a minimum of 63% pressure decay during diastole; thus, an RC_FIT_ analysis was applicable to 409 curves (84.3%) corresponding to 164 patients (90%). The coefficient of variation for R_SL_ and RC_FIT_ for consecutive heartbeats in each patient was 17.9 ± 16.2% for R_SL_ and 16.9 ± 14.6% for RC_FIT_.

The effects of reflection waves and heart rate on the quality of the curve fit was evaluated whereby significant impact of these two variables observed on curve fit (se supplementary material). Subsequent analysis was performed to compare the cases (*n* = 18) in which the RC_FIT_ analysis was not applicable to the corresponding group with obtainable RC_FIT_ (164). The group of patients without obtainable RC_FIT_ demonstrated significantly higher pulmonary artery pressure, PVR, and HR, as well as lower PAWP. No difference in CI was observed between the two groups (see supplementary material).

### Diagnostic significance

ROC analysis was performed for PAH and PH-LHD subgroups to investigate the diagnostic value of RC in differentiating between these two distinct hemodynamic states. RC_EST_ demonstrated a strong discriminatory ability [AUC = 0.86, SE = 0.03, *p* < 0.001, CI = 0.79–0.93; at the cutoff value of 0.53 s the sensitivity and specificity were 80% and 77%, respectively]. The corresponding values for RC_SL_ were slightly lower [AUC = 0.81, SE = 0.04, *p* < 0.001, CI = 0.72–0.89; cutoff value of 0.37 s, sensitivity and specificity 71% and 71%, respectively]. Finally, the RC_FIT_ displayed a weaker diagnostic capacity for identifying PAH [AUC = 0.7, SE = 0.06, CI = 0.57–0.82, *p* = 0.002), cutoff value of 0.29 s, sensitivity and specificity of 73% and 61%, respectively.

### Prognostic significance

In the PH-LHD subgroup, the prognostic significance of three different RC analyses was examined using Cox regression. Across a median follow-up of 630 days (IQR: 354–980), encompassing 53 primary outcome events (26 deaths, 28 heart-transplantation (H-tx) /left ventricular assist device implantation (LVAD)). RC measurements did not demonstrate significant prognostic value in univariate analysis (RC_EST_, *p* = 0.39; RC_FIT_, *p* = 0.85; RC_SL_, *p* = 0.88). Subsequent Cox regression analysis revealed no significant association between PAC measurements and the composite outcome of death or H-tx/LVAD (PAC_EST_: *p* = 0.187, PAC_SL_: *p* = 0.28, PAC_FIT_: *p* = 0.54).

## Discussion

This study represents the first attempt to investigate the validity of the available approaches for RC calculation in various states of pulmonary circulation. We compared the RC time estimates obtained using empirical, semilogarithmic, and direct fit analyses of the PA pressure decay. Our findings corroborate previous research showing that the empirical approach overestimates the RC time. Conversely, the semilogarithmic method provides reliable calculations, particularly with respect to groups of conditions. Moreover, we demonstrated that RC exhibits significant variations in different states of pulmonary hypertension (PH) and possesses substantial discriminatory ability in PH diagnostics.

The empirical approach for RC approximation overestimates RC values because of the systematic overrating of inherent pulmonary arterial compliance. Specifically, the calculation of PAC as the ratio of stroke volume (SV) to pulmonary arterial pulse pressure (PA_PP_) in the standard PVR × PAC equation fails to account for the fact that only a fraction of the right ventricular SV flows through the pulmonary arterial tree during systole, whereas the remaining portion is stored in the compliant arterial tree. Earlier studies have shown that the SV/PA_PP_ ratio overestimates PAC by 60–80% in animals [8] and 60% in patients with suspected PH [17]. Our results concur with the aforementioned observations, as we demonstrated that RC_EST_ generated approximately 60% higher values than RC derived from the semilogarithmic method (RC_SL_) and curve fit (RC_FIT_) in each of the three study subgroups.

In addition to the aforementioned physiological explanation, the extent of the discrepancy between the RC_EST_ and RC_FIT_values appears to be strongly associated with DPG levels, as evidenced by the findings of the present study. However, it should be noted that this association became insignificant at higher DPG values. The standard PVR equation relies on the pulmonary artery wedge pressure levels near those of pulmonary artery diastolic pressure for the PAWP to accurately represent the actual zero-flow pressure. However, this condition is valid only in patients without significant precapillary pulmonary involvement. In contrast, in conditions with evident changes in precapillary function, such as those characterized by high DPG values, zero-flow pressure is substantially higher than PAWP, resulting in significant PVR overestimation when employing the PVR equation [18]. Notably, we show that the association between DPG and RC_FIT_ became increasingly weaker at higher DPG values, which was not the case for the corresponding relationship with RC_EST_ and DPG. This indicates that at increasing levels of precapillary involvement, RC_FIT_ might yield misleading values, possibly because of the steep pressure decline, where the decay of pressure might not follow a monoexponential function (Fig. 1S).

Our study revealed that, in both the non-PH and PH-LHD patient groups, the RC_SL_ values closely matched the direct RC measurements obtained through curve fit analysis. However, in the PAH subgroup, the RC_SL_ yielded higher values than the corresponding curve fit measurements. This discrepancy may be due to the shape of the PAP curve in the PAH state, in which the curve fit may not optimally reflect the abrupt pressure decay occurring during early diastole in this patient group. The RC_SL_measurements in our study were similar to previously reported results [10, 19]. Despite the nearly identical values between the RC_SL_ and RC_FIT_ in the group analysis, the broad limits of agreement did not support the interchangeability of the two methods on an individual basis. Furthermore, the inability of the equation to calculate the natural logarithm for negative DPG values in the denominator limits the feasibility of RC_SL_ because of the frequent occurrence of negative DPG values in patients with PH-LHD.

The assumption of RC time constancy in health and disease has been refuted, as several studies have shown that RC_EST_ varies in different states of pulmonary circulation. First, Tedford et al. [4] and subsequently other groups [5, 20, 21] documented shorter RC_EST_ in PH-LHD compared to precapillary PH, an observation supported and extended by our findings, as we demonstrate that this discrepancy holds true even when curve fit-derived RC time was calculated. Furthermore, we showed that with regard to PH-LHD, CpC-PH was associated with a significantly longer RC time than IpC-PH, which can partly be ascribed to the stiffer vascular properties of CpC-PH [22]. Nevertheless, our results do not demonstrate any significant difference in RC between the PH-LHD and non-PH subgroups, which might be attributed to the fact that the PAWP elevation observed in PH-LHD is associated with a shorter RC time, as previously demonstrated [4].

RC showed a notable aptitude to distinguish PAH from the other two investigated hemodynamic states. Notably, among the three RC measurements, the empirical approach provided superior diagnostic information in this context. This might seem counterintuitive given the inaccuracies of the specific approach for RC estimation. However, this simplified RC analysis incorporates hemodynamic variables such as stroke volume and pulmonary artery pulse pressure, which may explain the superior diagnostic performance of the test. Additionally, constraints in RC_FIT_ and RC_SL_ analyses, as previously described, and the variability in the quality of curve fit may have contributed to the lower diagnostic capacity of these analyses.

Nevertheless, the diagnostic utility of RC is limited by the wide range of RC values within each hemodynamic state. Most published studies reported a coefficient of variation of > 30% within a given disease subgroup [1, 3, 4, 7], which is consistent with our results. In addition, we demonstrated that the degree of variation was comparable for all the three RC methods. Furthermore, we show a > 15% beat-to-beat variation in the RC time for both semilogarithmic and curve fit analyses. Regarding the valid question of whether the curve fit indeed follows a monoexponential function of time, our results show that in the vast majority of cases, the pressure decay during diastole acts monoexponentially as provided by the high degrees of goodness of fit (IQR = 0.85–0.97). However, as indicated by our findings, the occurrence of reflection waves and increased heart rate should be carefully considered because of their impact on the quality of curve-fit analysis.

Despite challenges associated with measurement variability and the varying behavior of RC time across different states of pulmonary circulation, larger-scale investigations are necessary to comprehensively evaluate its potential contribution to hemodynamic assessment. Furthermore, future research endeavors should explore the role of RC in aiding the classification of different HFpEF subtypes and in monitoring patients undergoing treatment for PAH.

### Limitations

Our study investigated the RC curve fit analysis using a monoexponential fit model. However, more complex fit models may occasionally provide a more accurate assessment of pressure decay. Importantly, the analysis of the curve fit requires adequate quality pressure recordings and reflection waves (see Supplementary Material). However, as demonstrated in our study, apart from the visual inspection of the PAP curve, a qualitative measure of curve fit can be used as an index to indicate the fit quality. In our study, RC failed to demonstrate significant prognostic information. However, it lacked statistical power for that purpose. Finally, in our cohort, we included patients with specific entities of pulmonary circulation; thus, the results cannot be generalized to other pathophysiological entities.

### Supplementary Information

Supplementary Material 1.

## Data Availability

The datasets used and/or analyzed during the current study are available from the corresponding author upon reasonable request.

## Abbreviations

CI: Cardiac index
CO: Cardiac output
CpC-PH: Combined pre-and postcapillary Pulmonary hypertension
DPG: Diastolic pressure gradient
EF: Ejection fraction
HFpEF: Heart failure with preserved ejection fraction
HFrEF: Heart failure with reduced ejection fraction
HR: Heart rate
IpC-PH: Isolated postcapillary Pulmonary hypertension
PAC: Pulmonary arterial capacitance
PAH: Pulmonary arterial hypertension
PAP: Pulmonary artery pressure
PAP_S_: Peak systolic PAP
PAP_D_: End-diastolic PAP
PAP_M_: Mean PAP
PAWP: Pulmonary artery wedge pressure
PVR: Pulmonary vascular resistance
PH: Pulmonary hypertension
PH-LHD: Pulmonary hypertension due to left heart disease
RC: Resistance and capacitance product during the diastolic period
RC_EST_: RC time derived using the empirical approach
RC_SL_: RC time derived using the semilogarithmic equation
RC_FIT_: RC-time derived using direct curve fit analysis
RHC: Right Heart catheterization

## References

[1] Lankhaar JW, Westerhof N, Faes TJ (2006). Quantification of right ventricular afterload in patients with and without pulmonary hypertension. Am J Physiol Heart Circ Physiol.

[2] Saouti N, Westerhof N, Postmus PE, Vonk-Noordegraaf A (2010). The arterial load in pulmonary hypertension. Eur Respir Rev.

[3] Lankhaar JW, Westerhof N, Faes TJ (2008). Pulmonary vascular resistance and compliance stay inversely related during treatment of pulmonary hypertension. Eur Heart J.

[4] Tedford RJ, Hassoun PM, Mathai SC (2012). Pulmonary capillary wedge pressure augments right ventricular pulsatile loading. Circulation.

[5] Dragu R, Rispler S, Habib M (2015). Pulmonary arterial capacitance in patients with heart failure and reactive pulmonary hypertension. Eur J Heart Fail.

[6] Grignola JC (2014). Is the time constant of the pulmonary circulation truly constant?. Eur Respir J.

[7] Tedford RJ (2014). Determinants of right ventricular afterload (2013 Grover Conference series). Pulm Circ.

[8] Segers P, Brimioulle S, Stergiopulos N (1999). Pulmonary arterial compliance in dogs and pigs: the three-element windkessel model revisited. Am J Physiol.

[9] Engelberg J, Dubois AB (1959). Mechanics of pulmonary circulation in isolated rabbit lungs. Am J Physiol.

[10] Reuben SR, Butler J, Lee GJ (1971). Pulmonary arterial compliance in health and disease. Br Heart J.

[11] Lang RM, Badano LP, Mor-Avi V (2015). Recommendations for cardiac chamber quantification by echocardiography in adults: an update from the American Society of Echocardiography and the European Association of Cardiovascular Imaging. Eur Heart J Cardiovasc Imaging.

[12] Vachiery JL, Tedford RJ, Rosenkranz S (2019). Pulmonary hypertension due to left heart disease. Eur Respir J.

[13] Borlaug BA, Nishimura RA, Sorajja P, Lam CS, Redfield MM (2010). Exercise hemodynamics enhance diagnosis of early heart failure with preserved ejection fraction. Circ Heart Fail.

[14] Humbert M, Kovacs G, Hoeper MM (2022). 2022 ESC/ERS Guidelines for the diagnosis and treatment of pulmonary hypertension. Eur Heart J.

[15] Liu Z, Brin KP, Yin FC (1986). Estimation of total arterial compliance: an improved method and evaluation of current methods. Am J Physiol.

[16] Chemla D, Lau EM, Papelier Y, Attal P, Herve P (2015). Pulmonary vascular resistance and compliance relationship in pulmonary hypertension. Eur Respir J.

[17] Muthurangu V, Atkinson D, Sermesant M (2005). Measurement of total pulmonary arterial compliance using invasive pressure monitoring and MR flow quantification during MR-guided cardiac catheterization. Am J Physiol Heart Circ Physiol.

[18] Kafi SA, Melot C, Vachiery JL, Brimioulle S, Naeije R (1998). Partitioning of pulmonary vascular resistance in primary pulmonary hypertension. J Am Coll Cardiol.

[19] Senzaki H, Kato H, Akagi M, Hishi T, Yanagisawa M (1995). New criteria for the radical repair of congenital heart disease with pulmonary hypertension. To avoid postoperative residual pulmonary hypertension. Jpn Heart J.

[20] DuPont M, Mullens W, Skouri HN (2012). Prognostic role of pulmonary arterial capacitance in advanced heart failure. Circ Heart Fail.

[21] Pellegrini P, Rossi A, Pasotti M (2014). Prognostic relevance of pulmonary arterial compliance in patients with chronic heart failure. Chest.

[22] Najjar E, Lund LH, Hage C (2021). The differential impact of the left atrial pressure components on pulmonary arterial compliance-resistance relationship in heart failure. J Card Fail.

