# Supplementary material for: Insights into RC time curve fit analysis of pulmonary artery pressure decay
Source: BMC Pulm Med. 2024 Jun 25;24:295. doi: 10.1186/s12890-024-03107-5 (PMC11197313; doi:10.1186/s12890-024-03107-5)
Supplement: Supplementary file 1 — Supplementary Material 1. [file 12890_2024_3107_MOESM1_ESM.zip › SUPPLEMENTARY final.docx]

SUPPLEMENTARY FILE

**Quality determinants for the curve fit**

The quality of curve fit displayed significant variations and was assessed using Mean Square Error (MSE). Figure 1S. The MSE for the entire study cohort was relatively low (1.4; 0.6-3.7). However, significant differences were observed among the three groups with the lowest values demonstrated in the non-PH group (MSE = 0.9; 0.4-2.3), followed by the PH-LHD group (MSE= 1.4; 0.6-3.5), while PAH subgroup exhibited the highest values (MSE=2.9; 0.8-9.1).

To investigate the factors influencing the MSE for curve-fit analysis, a regression analysis was conducted with log [MSE] as the dependent variable and DPG, reflection wave peak amplitude (P_REFL_), and heart rate (HR) as independent factors. The overall regression analysis revealed a statistically significant relationship, with the three factors collectively explaining 34% of the variance in the MSE (r^2^ = 0.34, F (3,160) = 26.8, p < 0.001). Further examination of the coefficients revealed that P_REFL_ (B = 0.07, β = 0.48, p < 0.001) and HR (B = 0.01, β = 0.25, p < 0.001) had a substantial impact on MSE. In contrast, DPG was not significantly associated with MSE (P = 0.99).

P_REFL_ values in patients with non-PH (2.3; 1.0-4.2 mmHg) and those with PH-LHD (2.2; 1.0-5.3 mm Hg) were lower than those in patients with PAH (3.2; 0.7-5.6 mmHg). As displayed in Figure 2S, the correlation between P_REFL_ and MSE was most pronounced in the non-PH group, followed by the PH-LHD and PAH groups. Notably, no significant association was found between P_REFL_ and the variations in RC measurements.

In terms of HR, within the entire cohort, HR < 80 beats/min (bpm) was recorded in 137 patients, whereas 45 patients demonstrated HR ≥ 80 bpm. Of the 164 RC_FIT_ analyses, the feasibility rate was 93% (n=127) in those with an HR < 80 bpm and 82% (n=37) in the corresponding group with an HR ≥ 80 bpm. Furthermore, the MSE for the curve-fit analysis was significantly lower in those with HR< 80 bpm compared to the subgroup with HR ≥ 80 bpm (2.3 ± 2.8 vs. 7.2 ± 10, p < 0.001).

Finally, in order to investigate potential disparities between the group of patients in whom the RC_FIT_ was obtainable as compared to the group without an applicable RC_FIT_ analysis the two groups were compared in regard to the hemodynamic variables. As revealed in the table below, the group of patients without obtainable RC_FIT_ was characterized by higher pulmonary pressures, higher PVR and HR and lower PAWP. The aforementioned findings indicate that in cases with pre-capillary PH particularly in conjunction with higher HR the curve fit analysis is less frequently feasible possible due to a combination of abrupt pulmonary

|  | **RC_FIT_ feasible (n=164)** | **RC_FIT_ unfeasible (n=18)** | **P-value** |
| --- | --- | --- | --- |
| **Hemodynamic data** |  |  |  |
| **CI** (L/min/m^2^) | 2.4 (1.9, 2.9) | 2.3 (1.8, 2.9) | 0.045 |
| **HR** (beats/min) | 68 (61, 78) | 75 (67, 86) | 0.043 |
| **PAP_M_** (mmHg) | 31 (25, 39) | 42 (32, 51) | 0.002 |
| **PAP_D_** (mmHg) | 20 (16, 24) | 31 (23, 39) | <0.001 |
| **PAWP** (mmHg) | 16 (13, 20) | 9 (7, 13) | <0.001 |
| **PVR** (WU) | 2.7 (1.7, 4.7) | 7.3 (4.6, 12.6) | <0.001 |

pressure decline as well as shorted diastolic time interval.

Supplementary Figures Legends

Figure 1S. Panel of figures illustrating the relationship between RC measured by the three different methods and diastolic pressure gradient (DPG). The study cohort was dichotomized into two subgroups: those with a DPG < 7 mmHg (blue dots) and those with an elevated DPG ≥ 7 mmHg (red dots). The left panel illustrates the relationship between the RC derived by curve fit analysis (RC_FIT_) and the DPG. The central panel illustrates the relationship between the RC derived by the semilogarithmic approach (RC_SL_), and the right panel shows the regression analysis between the RC derived using the empirical approach (RC_EST_) and the DPG.

Figure 2S. The figure illustrates three curve-fit analyses of different qualities. At the top, the curve fit displayed excellent quality with a low mean square error (MSE = 0.08). In the middle panel, the curve fit analysis displayed a lower degree of quality of fit (MSE=2.7). In the lower panel, the curve exhibits an even lower degree of fit (MSE=5.4).

Figure 3S. Regression plot between the peak amplitude of reflection waves (P_REFL_) and mean square error (MSE) for curve-fit measurements. Yellow dots signify the subgroup without pulmonary hypertension (non-PH), green dots denote the subgroup with PH due to left heart disease (PH-LHD), and red dots denote the subgroup with pulmonary arterial hypertension (PAH).
