# Supplementary figures and images for: Insights into RC time curve fit analysis of pulmonary artery pressure decay
Source: BMC Pulm Med. 2024 Jun 25;24:295. doi: 10.1186/s12890-024-03107-5 (PMC11197313; doi:10.1186/s12890-024-03107-5)

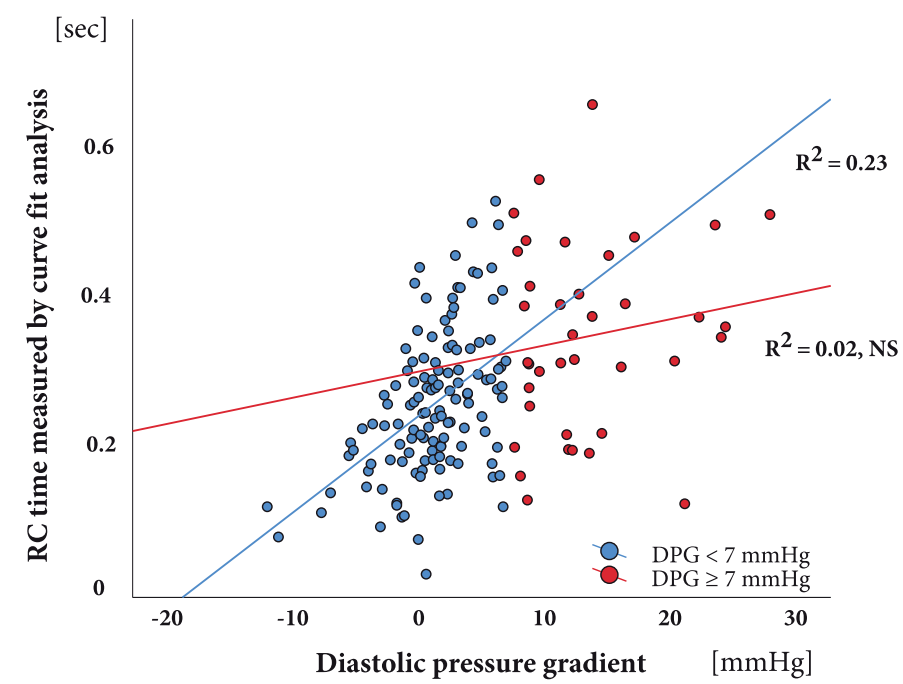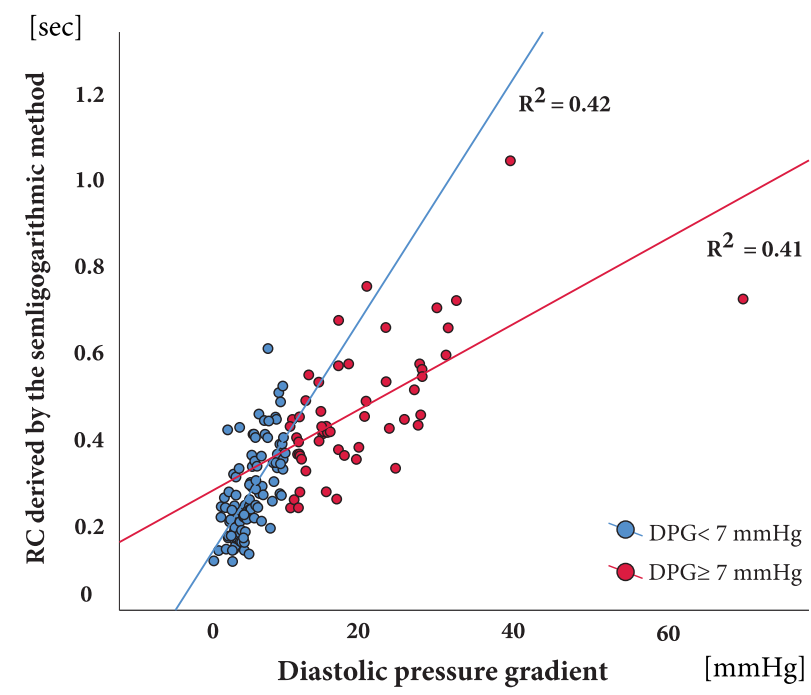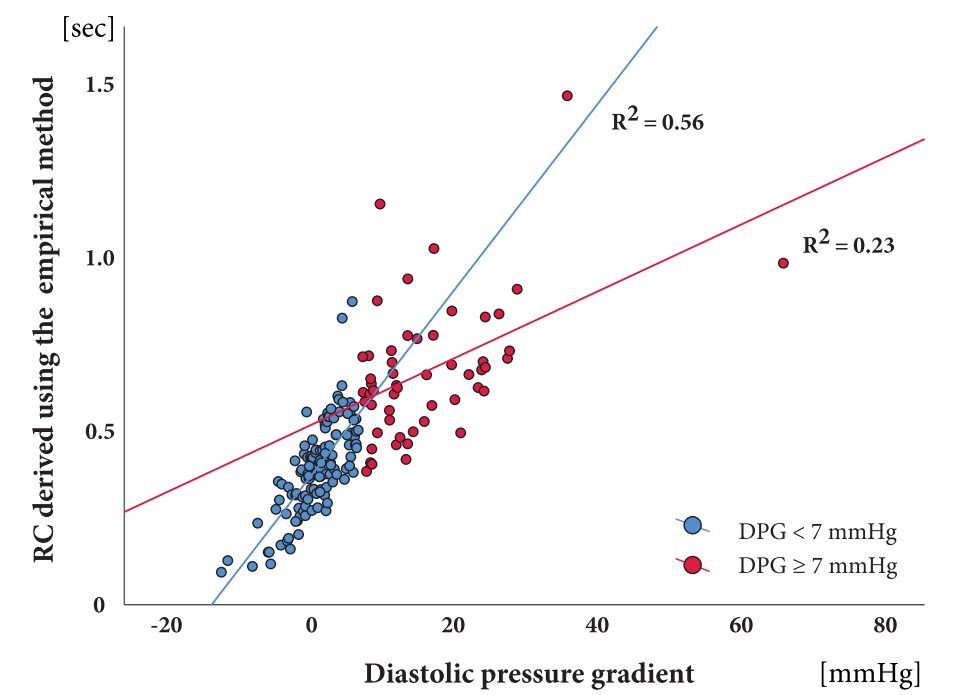

Supplement: Supplementary file 1 — Supplementary Material 1. [file 12890_2024_3107_MOESM1_ESM.zip › FIGURE 1S Panel plot DPG vs 3 RC.pdf]

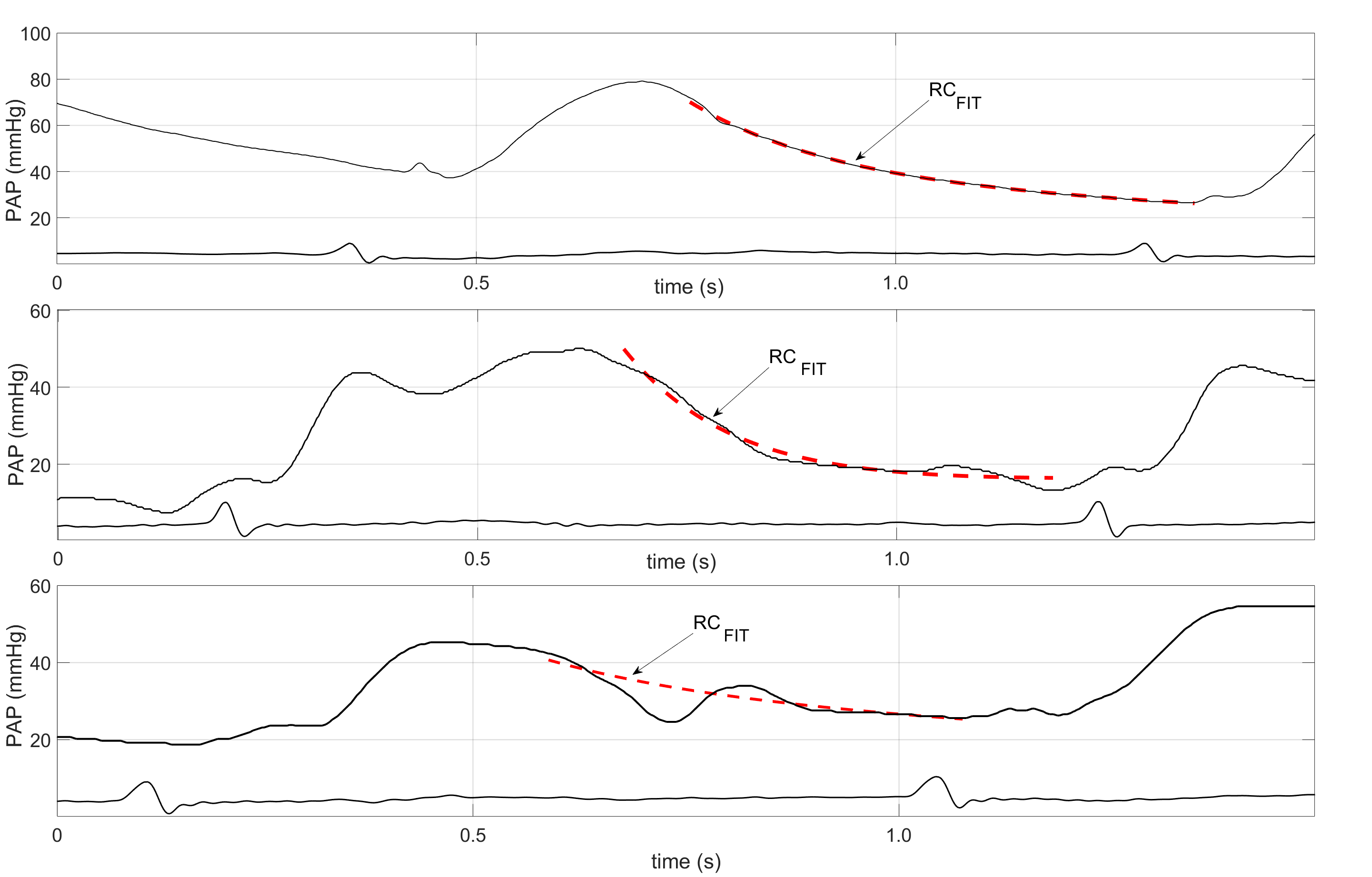

Supplement: Supplementary file 1 — Supplementary Material 1. [file 12890_2024_3107_MOESM1_ESM.zip › FIGURE 2S PANEL of 3 different RCfit.tif]

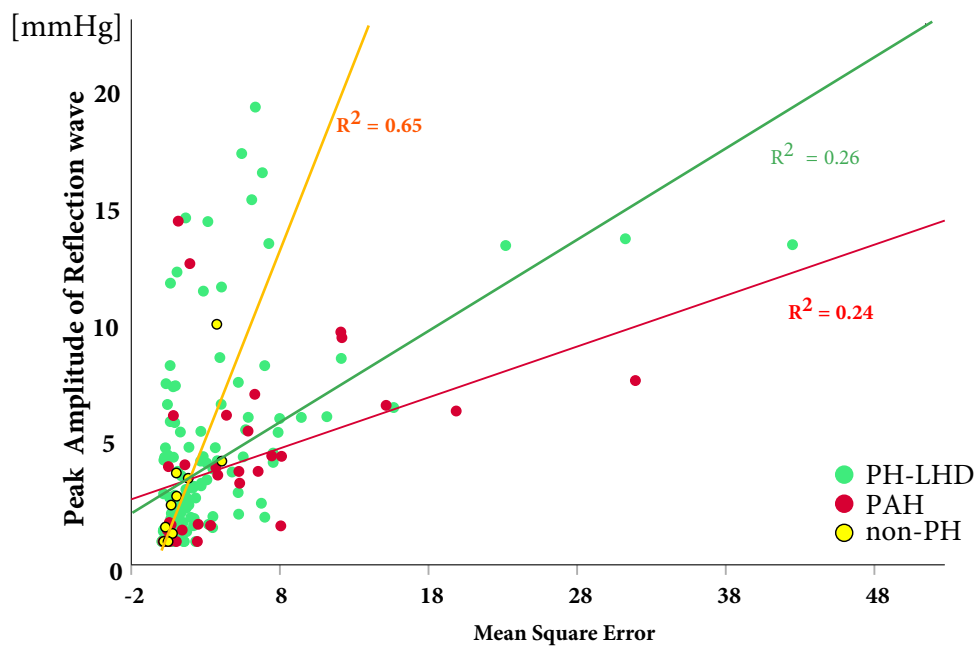

Supplement: Supplementary file 1 — Supplementary Material 1. [file 12890_2024_3107_MOESM1_ESM.zip › Figure 3S MSE vs Refle wave in 3 groups.pdf]
